# Active Learning of the HL7 Medical Standard

**DOI:** 10.1007/s10278-018-0134-3

**Published:** 2018-10-23

**Authors:** Rita Noumeir

**Affiliations:** Department of Electrical Engineering, École de Technologie Supérieure, 1100 Notre-Dame West, Montreal, QC H3C 1K3 Canada

**Keywords:** Active learning, Healthcare, Medical informatics, HL7 standard, Problem based, Learning, Interoperability

## Abstract

Health Level Seven (HL7®) is a standard for exchanging information between medical information systems. It is widely deployed and covers the exchange of information in several functional domains. It is very important and crucial to achieve interoperability in healthcare. HL7 competences are needed by all professionals touching information technology in healthcare. However, learning the standard has always been long and difficult due to its large breadth as well as to large and complex documentation. In this paper, we describe an innovative active learning approach based on solving problems from real clinical scenarios to learn the HL7 standard, quickly. We present the clinical scenarios used to achieve learning. For each scenario, we describe and discuss the learning objectives, clinical problem, clinical data, scaffolding introduction to the standard, software used, and the work required from the students. We present and discuss the results obtained by implementing the proposed approach during several semesters as part of a graduate course. Our proposed method has proven that HL7 can be learned quickly. We were successful in enabling students of different backgrounds to gain confidence and get familiar with a complex healthcare standard without the need for any software development skill.

## Introduction

The Health Level Seven (HL7®)^1^ standard is very important to achieve interoperability in healthcare. It is widely used for communicating medical information between various information systems [1]; it is therefore a cornerstone to implement the Electronic Health Record (EHR). EHR improves healthcare decisions by allowing access to the patient’s relevant clinical information at the decision-making point. EHR is a distributed system that results from the interactions and cooperation of various independent information systems to achieve a specific healthcare process. Therefore, deploying EHR requires the successful exchange of information between several systems. Without HL7, there is no interoperability and no EHR.

Although HL7 is crucial for achieving EHR, learning it has been achieved ad hoc, after long exposure to interoperability problems, sometimes combined with specialized training provided outside of the academic structure. By introducing HL7 in a graduate course on distributed systems in healthcare, we have faced several problems. These problems are the following: (1) the domain is multidisciplinary, (2) the breadth of required healthcare processes knowledge is large, (3) the standard documentation is huge and formal introductory texts are not available, (4) the students’ background is extremely diversified.

HL7 is at the intersection of healthcare, engineering, and Information Technology (IT). It covers almost all functional domains encountered in healthcare including patient management and administration, order management, and observation reporting. In order to achieve interoperability, many other standards are needed. Some are not specific to healthcare such as the ones that pertain to security or to indexing. Others are specific to healthcare such as the Digital Imaging and Communication in Medicine (DICOM). All these standards are used in practice. Their cooperation is defined either site-specifically, or according to formal international integration profiles described by the Integration Healthcare Enterprise (IHE) [2, 3].

Several versions of the HL7 standard exist. HL7 v2 is largely implemented and deployed in almost every healthcare hospital or clinic. HL7 v3 is adopted by many governments and agencies as a standard required for EHR and is used in many of the IHE integration profiles [3]. HL7 has very recently proposed a new framework, the Fast Healthcare Interoperability Resources (FHIR®), which combines features of HL7 v2 and HL7 v3 with the latest web technologies such as the Representational State Transfer (REST) architecture to facilitate implementation. All versions co-exist and it is common to have several versions of the standard deployed simultaneously and cooperating at the same institution.

HL7 is needed by almost every professional involved with healthcare processes. Knowing HL7 is needed by engineers not only to design and implement interoperable medical systems but also to maintain them. It is needed by clinicians and IT specialists to implement healthcare workflows. Administrators need HL7 to manage the purchase processes of such systems. Companies need HL7 to develop, test, and deploy healthcare systems. It is needed by hospitals and clinics to purchase their clinical applications and to manage and maintain them. HL7 is also needed by governmental agencies so they can provide specifications and regulations to enable the integration of healthcare information at the regional and national levels.

This large range of dependence on HL7 is reflected in the students’ background. Their prior experience and training include engineering, IT, medical technologies, biology, nursing, and medical practice. All rely on HL7 to accomplish a specific purpose whether system design or writing a request for purchase. Some students have extensive programming skills while others have limited computer skills. All have the same objective to achieve better healthcare and mastering HL7 is a must for all of them.

We are interested in enabling students with diversified backgrounds to quickly learn and use HL7.

The standard documentation is written to be a complete reference, but not necessarily to be easy to read or to be used for quick learning. The HL7 v3 documentation is structured and constructed to be mainly navigated using a browser. The message names as well as the names of the other artifacts are very hard to be remembered by a human. The presence of hyperlinks allows non-sequential navigation; however, it is very common to get lost especially for a novice reader. Other resources and documentations, such as the primer book [4], concentrate on the information model along with some general concepts. They are not very helpful for quickly implementing something useful with the standard, such as exchanging information.

On the other hand, active learning is increasingly attracting interest as it enhances learning [5]. Amongst the various active learning strategies [6], problem-based learning appears to be well adapted to help us achieve our pedagogical goals in a short time, mitigating the difficulties discussed above.

In this paper, we describe a problem-based strategy to learn the HL7 standard. Following the method of [7] to guide the design of the learning activities, we present and discuss the problem definition, and the support provided to the students.

In the next section, we present our method: problem definition in terms of learning objectives, clinical scenarios, and required work as well as resources to support the learning activities such as clinical data, scaffolding documentation to navigate the standard text, and validation software. The software used by the students was developed specifically to help those with no software development experience, get familiar and use HL7 v2 and v3 in a short time. Results from our experience teaching both versions of the HL7 standard are presented and discussed in the “Results” section. We also describe students’ interests and results.

## Method

First, we defined the problems that enable achieving the learning outcomes and engage the students in activities presenting challenges from the real world. We defined two complex problems articulated around clinical scenarios. The first scenario is taken from a radiology environment and is used to learn HL7 v2. The second scenario is taken from an EHR environment and is used to learn HL7 v3. In addition to the main learning outcome, which is getting familiar with the HL7 standard, we articulated the problems to achieve secondary larger objectives: (1) introducing interoperability, (2) and getting familiar with EHR workflows.

Second, we developed the support material to enable active learning. Several criteria have guided this development: (1) the learning objectives need to be achieved by students with no programming skills; therefore, we have developed a complete software infrastructure allowing students to concentrate strictly on data and information. (2) Students’ confidence regarding the navigation of the complex and large standard documentation needs to be increased; therefore, we have developed a scaffolding document describing how to read and navigate strictly those parts of the standard needed to successfully solve the problems. This is the opposite of how normally HL7 is presented as we did not cover the complete data model. (3) Students’ confidence regarding their ability to successfully complete the educational work needs to be increased; therefore, we developed validation software that provided feedback to students and helped them self-assess their work.

In the following sub-sections, we present the clinical scenarios. For each scenario, we describe and discuss (1) the learning objectives, (2) the clinical data provided to the students, (3) the scaffolding introduction to the HL7 standard, (4) the work required from the students, and (5) the software that was provided to the students to help them achieve their work.

### Clinical Scenario: Generating an Order and an Observation Message for an Imaging Acquisition

A typical scheduled radiology image acquisition is performed upon receiving an order from an order placer system. The order message is an HL7 Order Message (ORM) that communicates the patient demographics and the ordering physician information, as well as information about the imaging procedure to be performed. The ORM is received by the Radiology Information System (RIS) that generates a modality work item on its worklist. When the imaging acquisition equipment is integrated with the RIS, a modality worklist query, using the DICOM standard, is sent from the acquisition equipment to the RIS in order to obtain a list of imaging acquisition steps to perform. The human operator at the acquisition equipment console typically chooses one item from the list and performs the imaging acquisition on the patient. It results in the generation of a DICOM image whose patient’s demographic and procedure information are automatically copied from the modality worklist fields into the DICOM image header. The worklist fields are initially copied from the received ORM. The mapping between the ORM fields and the image DICOM header fields are detailed in the IHE technical framework [2].

After the acquisition is completed, the radiologist interprets the image and generates a diagnostic report [8] that may be communicated to a third-party system by means of an HL7 Observation Result (ORU) message. In addition to the patient’s demographic and procedure fields, the ORU message contains the impressions and interpretation of the radiologist.

**Fig. 1:**
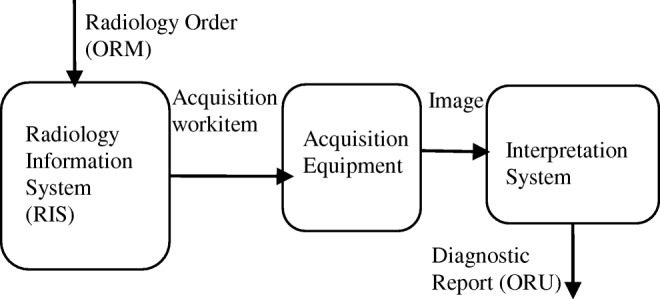
Radiology process data flow

The data flow of this simplified radiology process is depicted in Fig. 1. The acquisition step needs the patient’s demographics and procedure information in order to generate an image. The interpretation step needs the image in order to generate the results. The order, the image, and the result all correspond to the same radiology process and belong to the same patient. Therefore, they need to contain the same patient and procedure information.

#### Learning Objectives

The objective is to get familiar with the HL7 v2 message structure as well as with the mapping of information between DICOM and HL7. This later objective introduces the student to the larger domain of interoperability and the consistency of information. It also enables the consolidation of previously acquired skills from learning DICOM for example.

#### Clinical Data

We provide a de-identified DICOM image to the students. We ask them to generate:

1. the order message that could have been used to request the acquisition of that image and
2. the observation message that could be generated as the result of the interpretation process of that image.

Students have to use the patient’s and procedure information from the provided image.

#### Scaffolding Introduction to HL7 v2 Standard

After pointing to the students where to get the standard documentation [9], we explore all functional domains covered by the standard by quickly browsing its various chapters. Following this overview, a sample message—registering a new patient—is examined in detail in order to grasp the encoding syntax: segments, segment delimiter, segment name, special header segment (MSH), special event segment (EVN), fields, field separator, and field numbers. The Patient Name field (PID.5) is studied: its position in the segment, its data type, its components, and the component separator. Sub-components and their separator are introduced by examining an example of an observation request segment (OBR) and by studying the Principal Result Interpreter field (OBR.32). Field repetition is introduced with an example of an Observation Value field (OBX.5), whose type is text. The message structure is introduced by exploring the Register a Patient message (ADT-A04). Special attention is given to the MSH segment and its fields. The response message is introduced and the structure of the acknowledgement message (ACK) is detailed. Special attention is given to acknowledgement (MSA) and error (ERR) segments and their fields. This quick introduction is achieved by browsing the standard documentation and by analyzing samples of real messages.

#### Required Work

Using a simple text editor or a specialized XML editor, students are required to provide well-formed HL7 ORM and ORU message in a radiology service request context. They have to manually write both messages using information available in the header of a single DICOM image that is provided to them. The messages generated by students need to contain semantically meaningful information that is consistent with the medical image. Students can use the software that is also provided to them in order to validate their work and to correct any data inconsistency that is detected by the validation software. Solving this problem enables the students to get very familiar with the HL7 v2 message structure as well as with the mapping of information between DICOM and HL7 that is described in the IHE Radiology Technical Framework.

#### Validation Software

We provide the students software that was built specifically to help them succeed with the required task. The software is built using open-source libraries. The goal of the software is to validate the students’ work and to provide them feedback about their errors so they can correct them.

In order to validate the students’ work, the software (1) takes as input a DICOM image, a text file containing the order ORM message, and a text file containing the result ORU message; and (2) verifies, in addition to the HL7 message syntax, the content of the messages according to the mapping as discussed and specified in the IHE technical framework.

The software uses an open-source library to transform HL7 messages into XML documents; it also uses the open-source DCM4CHE library [10] to transform the DICOM image header into an XML document; with the Xpath technology used to query specific fields from the transformed XML documents, the software compares data and verifies the consistency of information.

The software is executed by the students as a simple command that takes three input files in a specific order: the HL7 messages and the image. It displays the result of the verification to the students in a tabular form where each row contains the data that is supposed to be consistent from the three sources. Obviously, to succeed in this work, students do not need any software development skills.

### Clinical Scenario: Registering and Searching for a Patient

EHR improves the quality of care mainly because it makes available relevant patient’s clinical information, when needed, to help in the diagnostic and treatment process. Accessing the patient’s record starts by identifying the patient which is achieved by searching for that patient using his/her demographic data including name, birth date, or identification number. A patient may be identified with several identification numbers, when known to a multi-site facility or at a regional level. Therefore, the EHR infrastructure comprises a system that is responsible for identifying a patient described with a combination of demographic parameters or with an identification number local to a specific institution. Identifying a patient requires querying this system using a HL7 v3 transaction. To fulfill this use case, other HL7 transactions are needed in order to ensure that the information within this patient registry is correct: a new patient needs to be registered with this patient registry where all the patient’s information is communicated; if an error is discovered in the patient’s demographic information after a patient is registered, an update transaction is used to correct it.

#### Learning Objectives

The objective is to become familiar with the HL7 v3 message structure and documentation as well as with several IHE integration profiles that are defined and detailed in the IHE Infrastructure Technical Framework, more specifically, the integration profiles that are needed to search for a patient in an EHR.

#### Clinical Data

We provide the students a database loaded with a few simulated patients’ demographics.

We ask them to register a patient and to correct the patient’s information afterwards. We also ask them to query for a patient’s demographic information and identifications.

#### Scaffolding Introduction to the HL7 v3 Standard

We point out to the students how to acquire the latest version of the HL7 standard [9].

The standard documentation is so large that one can get quickly lost. We introduce a top-down approach to navigate it: the functional domains are presented first; then we study a specific message in detail; we analyze its structure and we examine the data types that compose the message. We do not explore the complete data model nor all data types; to the contrary, we cover only what is needed to construct a specific message.

Our strategy is to learn the standard by constructing a message. However, it is important to know about the existence of the chapter entitled “Version 3 Guide.” It defines all the concepts used in the HL7 standard v3. One can get back to it when a detailed definition or explanation is needed.

The HL7 standard v3 defines messages that are used to exchange information in many domains such as patient administration, pharmacy, laboratory, immunization, reimbursements, reporting, and scheduling. Each domain is covered by a chapter. All chapters are grouped under the part “Universal Domains.” Students are encouraged to browse this part to get familiar with the HL7 coverage. The chapter “Patient Administration” defines several messages to register a new patient and to update the demographic and identification information of an already-registered patient. This is the chapter that we use to introduce the HL7 structure, messages, and data model.

The data model used in all interactions related to the administration is detailed in the Domain Message Information Models (D-MIM). To construct a message, a D-MIM is not needed in its entirety. We encourage the students to come back to this section and search for the definition of concepts and their relationships, when needed.

We are interested in registering a new patient. It is the Patient Activate (PRPA_RM201301UV01) message described within the Patient Topic Chapter. Obviously, the name of this artifact is not human friendly. The Patient Topic chapter has a common structure: Storyboards, Application Roles, Trigger Events, Refined Message Information Models (R-MIM), Hierarchical Message Descriptions (HMD), and Interactions.

Only R-MIM and HMD are needed for constructing a message. The R-MIM of interest is the one related to the message Patient Activate; it is a subset of the domain model D-MIM. It defines the classes, the attributes, and the associations needed to construct the message of interest. The HMD constitutes the content of the message content or its payload. It can be represented in various equivalent ways: graphical, tabular, or XML schema.

At this stage, students are encouraged to build a message using a specialized XML editor and the message schema.

To send a message, it needs to be wrapped with control and transmission information. The transmission wrapper includes information to assemble and send the interaction to a receiving application. It also specifies the level of acknowledgement that is expected from the receiving system. We focus on the levels recommended by IHE: the accept-level acknowledgement and the immediate response for a query response. By constructing the payload, the control act, and the transmission wrapper, the message is ready to be sent. Several ways for sending a message are defined by HL7 in the Transport Specifications chapter.

#### Required Work

We provide the students a software that consists of two components: (1) a server able to receive HL7 v3 messages for managing patients such as registering a new one or updating the information of an already-registered one; it also responds to patient-related queries; (2) a utility software that can be launched with a command line; it takes a file that contains an HL7 v3 message, sends its content to the server, and receives and displays the answer from the server.

The server is connected to a database pre-loaded with information about a few simulated patients. We ask the students to write well-formed HL7 messages. They are directed to use documentations including the HL7 v3 and the IHE technical framework, more specifically the Patient Demographics Query (PDQ) and the Patient Identifier Cross-Referencing (PIX) integration profiles [3]. Students have to execute a certain number of operations:

- Registering a new patient in a first hospital by sending to the server a Patient Registry Recoded Added message.
- Registering the previously registered patient in a second hospital, therefore using a patient’s identification (ID) different than the one used in the first registration.
- Correcting the patient’s name by sending the correct information to the server with a Patient Registry Recoded Revised message.
- Querying the server using the patient’s ID of the first hospital with the goal of retrieving that patient’s ID in the second hospital; this is done by sending the Patient Registry Get Identifier Query message.
- Retrieving the complete demographic patient’s data by querying the server with a subset of demographics information; this is done by sending a Patient Registry Find Candidate Query message.

Students are required to construct the HL7 message for each operation and to encapsulate it in a SOAP envelope that specifies the target and reply destinations as well as the action being performed.

#### Validation Software

We provide the students a system based on a client-server architecture, developed specifically for helping them execute and succeed at this task [11]. The students construct their HL7 message as an XML file; they use the client part of the software to send their message to the server. The response received back from the server is stored in a file to be further analyzed by them. Students can use any text or specialized XML editor to construct their message content. The content needs to be a valid HL7 v3 structure according to the schema of the intended message. Moreover, students need to wrap the HL7 content with a Simple Object Access Protocol (SOAP) envelop. The client software sends the file content to the server using HTTP.

The server receives HL7 messages to manage patients by either registering a new one or updating the demographic information of an already-registered patient. The server can also respond to demographic queries about patients or to patients’ identification queries. Therefore, the server acts as an IHE PDQ supplier and an IHE PIX Manager. Upon receiving a message, the server validates it and responds accordingly. It is easy to install and run.

The server was originally implemented to test the IHE PIX and PDQ v3 integration profiles [12, 13]. A database that stores information about patients has been added to the testing software. Querying information from the database enables the server to respond to HL7 queries. We pre-loaded the database with information about several simulated patients, and we provided the students with a web interface to visualize that information. The server receives an HL7 message and validates it. The validation capabilities and rules are the same as the ones used for testing the IHE profiles.

We have used open-source libraries to develop and assemble the software provided to the students. The open-source libraries and the developed software are available for multiple platforms as they are written in the Java language.

## Results

We set up and taught a graduate course to students with major in healthcare and in information technology. A total number of 76 students undertook the work, over several semesters. Students’ background included several engineering branches in addition to non-engineering such as biochemistry, physiotherapy, kinesiology, and radiology technology. Students with background in civil engineering as well as non-engineering students had fewer skills in computers as compared to the others. However, they had other skills such as project management, experience with patients’ interactions, or healthcare settings. One assistant provided supervision during nine hours distributed over few weeks, to help students in their work.

We asked the students to provide their HL7 messages for evaluation. We also asked them to analyze and comment on the communication received from the server, as responses to their messages.

Students worked on problems from real clinical scenarios.

The first problem is inspired from a common real radiology process. By working on this problem, students not only dealt with almost all information and data flavors involved in radiology but also had the chance to touch the DICOM standard. Most importantly, they could grasp how the HL7 and DICOM standards fit one relative to the other, how they complement each other, and how they work together. Furthermore, the skills students acquired went beyond learning one or multiple standards: they learned how standards can cooperate to achieve an integrated workflow. Integration in medicine is still a big challenge [14, 15], at the conceptual level and at the implementation one, as well. Becoming familiar with these challenges and with existing integration solutions is one of the most important skills students must acquire.

The second problem is inspired from the EHR where querying for patient’s identifier is a common need. EHR deployment has taken place in several countries. Its deployment is expected to expand as the quality of care is known to improve with EHR. Moreover, by reducing the duplication of tests and by improving the productivity, EHR is expected to increase healthcare efficacy and efficiency.

The interactions implemented as part of this problem are amongst the basic interactions needed to implement an EHR. In fact, a physician normally needs to access all the patient’s diagnostic information. However, part of this information may have been generated in a different hospital using a different patient’s identifier. Therefore, mapping several identifiers belonging to the same patient is a common need. On the other hand, the patient’s identifier is not always known. Instead, using only a subset of the patient’s demographic information for identification, such as using his/her name and/or birth date, is also a common need.

Therefore, skills learned from this work encompassed the HL7 standard and extended to reach the introduction to EHR sub-systems, their interactions, and the healthcare distributed process in general. Skills included several aspects related to HL7 such as constructing messages, understanding HL7 responses and acknowledgements, and getting familiar with typical HL7 interactions and workflows, as well as understanding the HL7 documentation structure and its navigation. Skills also included several IHE profiles and actors such as understanding the roles of the IHE PDQ supplier and PDQ consumer, the IHE PIX manager, and PIX consumer, as well as being introduced to the IHE documentation. Students also acquired skills related to XML and its schemas, an important tool in the domain of information technology.

Student’s opinion was gathered with a Likert-type scale questionnaire that is used by our university for every course. It consists of a set of statements on various aspects of their learning experience. We present in the first column of Table 1, the statements that are relevant to our method. To evaluate each statement, the student can choose from a scale of 1 to 5, where 5 means fully agree and 1 fully disagree. In Table 1, we also present the number of answers, the mean value, and the standard deviation (Std) for each statement.

**Table 1:** Results of students’ evaluation

Statement		Number	Mean	Std
1	The course is relevant for my professional training	54	4.2	0.8
2	The course is well adapted to my previous knowledge	51	4.1	0.9
3	The course content reflects the latest domain development	56	4.4	0.8
4	The workload is adequate with regard to the number of credits	57	4.1	0.9
5	The practical exercises allow to better understand and to apply the course content	57	4.3	0.9
6	Concrete examples are used sufficiently	57	4.4	0.8
7	The teaching material is adequate	57	4.5	0.8
8	Disparities between the students’ previous experiences are taken into account	55	4.5	0.7

The scores for statements 2 and 4 show that some students believe they lacked adequate previous knowledge and that the workload was higher that normally expected for a 3-credit course. The high score of the other statements, particularly for statements 7 and 8, shows that the material presented in this paper successfully helped students with their learning. We measured the reliability of this conclusion by calculating the Cronbach alpha coefficient, which has the value of 0.9835. This value (greater than 0.9) confirms excellent reliability and internal consistency of the statements.

Students were also given the chance to comment on the course. Very few comments were stated. Two persons proposed visiting a hospital to see the information and workflow in real life. This will be certainly considered for future improvement. Two persons complained about the heavy workload and questioned whether a prerequisite course would be adequate. One person stated that this course encouraged him/her to do research, and two stated how satisfied they were.

Students were motivated. At the beginning of the course, engineering students expressed interest in the human aspect of the problems while non-engineering students were interested in the increased career opportunities from acquiring skills related to interoperability. They succeeded in accomplishing the requested work except two. Both were not engineers. One abandoned quickly after realizing she was not able to dedicate enough time to succeed due to other responsibilities. The second abandoned because she lacked basic computer skills such as working in a client-server environment or running application from the console. To succeed, students dedicated more time than formally allocated. Motivation was sustained by their interest and by their confidence in their ability to achieve the requested work.

## Conclusion

We have presented a new method to actively learn HL7 rapidly, by solving real clinical problems. We have shown that working with real information objects and constructing messages that could have been exchanged in real clinical contexts enabled effective learning of our complex subject. We have also shown how solving a real problem can accelerate the learning and motivate the browsing and the searching of information in the very large standard documentation. In fact, to solve the practical problems, students browsed the standard documentation: to find the answers they needed, to construct the message, to find out and understand the message content and structure, and to find out and understand specific data types along with their meaning.

Concentrating on a specific problem directed the students to focus on a subset of the huge data model, helping them grasp that subset in detail without getting lost or intimidated by the size of that data model. Navigating around the standard to solve their problem helped them gain confidence and quickly grasp the structure of the standard itself. The students could not pretend to become experts in HL7; however, they can surely understand and participate in the design, implementation, and evaluation of information exchange using the HL7 standard.

We have presented an introduction guide to the standard that offers a more effective alternative to a bottom-up approach, learning from first principles. This introduction consists of a guided tour through the complex standard documentation. Presenting the documentation structure to easily navigate it provides enough confidence to search for the needed information. The reading guide and the problems to solve motivated the students to search for answers, enabling thus getting familiar with the documentation easily and rapidly.

We presented the software tools used to help the learning of the syntax, the semantics, and the functions of the HL7 standard, so software development is not needed. The software tools allow to send, receive, and validate messages generated for learning. Specialized XML editors or simple text editors to write HL7 messages are the only needed applications to accomplish the required learning work.

We believe our method for actively learning the HL7 standard can be used by anyone to quickly gain knowledge of this standard.

## Footnotes

1 HL7 and FHIR are the registered trademark of Health Level Seven International
